# Macrophages Characterization in an Injured Bone Tissue

**DOI:** 10.3390/biomedicines10061385

**Published:** 2022-06-11

**Authors:** Krisztina Nikovics, Marjorie Durand, Cédric Castellarin, Julien Burger, Emma Sicherre, Jean-Marc Collombet, Myriam Oger, Xavier Holy, Anne-Laure Favier

**Affiliations:** 1 Imagery Unit, Department of Platforms and Technology Research, French Armed Forces Biomedical Research Institute, 91223 Brétigny-sur-Orge, France; cedric.castellarin@intradef.gouv.fr (C.C.); emma.sicherre@supbiotech.fr (E.S.); myriam.oger@intradef.gouv.fr (M.O.); anne-laure.favier@intradef.gouv.fr (A.-L.F.); 2 Osteo-Articulary Biotherapy Unit, Department of Medical and Surgical Assistance to the Armed Forces, French Armed Forces Biomedical Research Institute, 91223 Brétigny-sur-Orge, France; marjorie1.durand@intradef.gouv.fr (M.D.); jean-marc.collombet@intradef.gouv.fr (J.-M.C.); 3 Microbiology and Infectious Diseases Department, French Armed Forces Biomedical Research Institute, 91223 Brétigny-sur-Orge, France; julien.burger@intradef.gouv.fr; 4 Department of Platforms and Technology Research, French Armed Forces Biomedical Research Institute, 91223 Brétigny-sur-Orge, France; xavier.holy@intradef.gouv.fr

**Keywords:** macrophages, hybridization chain reaction (HCR), cryosection, bone, cytokines, Masquelet induced membrane

## Abstract

Biomaterial use is a promising approach to facilitate wound healing of the bone tissue. Biomaterials induce the formation of membrane capsules and the recruitment of different types of macrophages. Macrophages are immune cells that produce diverse combinations of cytokines playing an important role in bone healing and regeneration, but the exact mechanism remains to be studied. Our work aimed to identify in vivo macrophages in the Masquelet induced membrane in a rat model. Most of the macrophages in the damaged area were M2-like, with smaller numbers of M1-like macrophages. In addition, high expression of IL-1β and IL-6 cytokines were detected in the membrane region by RT-qPCR. Using an innovative combination of two hybridization techniques (in situ hybridization and in situ hybridization chain reaction (in situ HCR)), M2b-like macrophages were identified for the first time in cryosections of non-decalcified bone. Our work has also demonstrated that microspectroscopical analysis is essential for macrophage characterization, as it allows the discrimination of fluorescence and autofluorescence. Finally, this work has revealed the limitations of immunolabelling and the potential of in situ HCR to provide valuable information for in vivo characterization of macrophages.

## 1. Introduction

Macrophages have an essential role both in osteoblast-mediated bone formation [1] and in osteoclast development [2,3], but the detailed function of these cells is not yet fully understood. In addition, the cytokines and other soluble factors secreted by macrophages can induce bone formation in vitro and in vivo [1,4,5,6,7,8]. 

**Figure 1:**
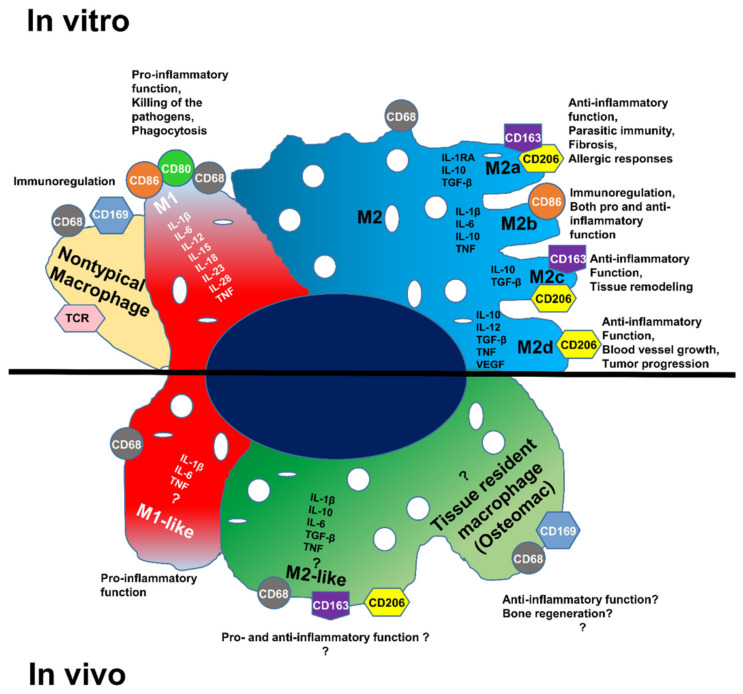
Macrophage polarization subtypes; different cytokine expressions and functions of the macrophage populations in vitro and in vivo.

For a long time, in vitro cultures were used to study the phenotypic characterization of macrophages as a model to control the extracellular environment [9,10,11]. In vitro macrophages can be classified into two families: (i) M1 macrophages and (ii) M2 macrophages. The M1 family expresses pro-inflammatory cytokines, such as tumor necrosis factor (TNF), interleukin-1 beta (IL-1β), interleukin-6 (IL-6), interleukin-12 (IL-12), interleukin-15 (IL-15), interleukin-18 (IL-18), interleukin-23 (IL-23), and interleukin-28 (IL-28) mediating inflammation. The M2 family expresses anti-inflammatory cytokines, such as interleukin-10 (IL-10), interleukin-1β receptor antagonist (IL-1RA), transforming growth factor-beta (TGF-β) and proangiogenic cytokines (vascular endothelial growth factor (VEGF) that resolve inflammation and also modulate the extracellular matrix (ECM). In vitro studies have shown that the M2 family characterization is more complex and can be subdivided into four subtypes: M2a, M2b, M2c, and M2d [12,13,14,15,16]. Each subtype expresses a distinctive panel of cytokines and plays a different role in tissue regeneration (Figure 1) [17,18,19,20,21,22,23]. Surprisingly, M2b and M2d macrophages also express pro-inflammatory cytokines including TNF, IL-1β, IL-6, or IL-12 [16,24]. This division is not perfect because specific (nontypical) macrophages do not belong to either group but play an important immunoregulatory role. An in vitro approach does not allow the study of other immune cells and their respective secreted cytokines normally present at the site of the regeneration. Indeed, the host response in vivo is more complex highlighting the difficulty to deduce in vivo results from in vitro observations. The knowledge of macrophage phenotypes under in vivo conditions is still poorly understood and further investigations are essential, especially in our case, to study macrophage involvement during bone regeneration. Accordingly, in vivo macrophages are named M1-like and M2-like macrophages or resolving macrophages (Figure 1) [23,25,26,27,28,29,30].

An additional category of macrophages called “Tissue-resident macrophages” is thought to participate in bone repair. Contained in almost all tissues, they are called ‘osteomacs’ when localized in the bone [1,2,31,32].

Biomaterial-based therapy is a useful method to improve bone regeneration; however, its underlying repair mechanism is not yet elucidated [33,34,35,36,37,38,39,40]. About 30 years ago, the French surgeon Alain-Charles Masquelet developed a new technique to repair bone defects called the Masquelet induced membrane [41]. The surgeon places a fixator along the bone and completes its missing parts with a biomaterial, an inert polymer that has been engineered for interacting with biological systems, usually polymethylmethacrylate (PMMA). Later on, a membrane called the Masquelet induced membrane will be generated all around the biomaterial as an immune reaction against a foreign body. It is essential for further bone regeneration [41,42,43]. In the literature, biomaterials are described to induce the appearance of the macrophages at the bone injury site [1,7,31,32,44,45,46].

One approach to characterize macrophages is the identification of their expressed cytokines. Different techniques, such as Northern blot, qPCR, microarray, flow cytometry analysis, and next-generation deep sequencing methods can be used. These methods only provide a result from a mixture of different cell types. In addition, measuring the expression level of the cytokines is not sufficient; the localization of the cytokine-expressing cells in the tissue should also be determined [47].

To localize the cytokine-expressing cells in the tissue, different methods are available: (i) expression of reporter constructs, but the limitation of this technique is the requirement of a transgenic animal; (ii) classical immunostaining techniques using antibodies against markers to detect specific proteins of the macrophages. However, since cytokines are generally secreted, it is difficult to determine exactly which cells produce this protein or peptide [48]. This technique can only distinguish between M1-like and M2-like macrophages. However, it is not appropriate for identifying M2-like subtypes, as there is currently no cell surface marker available to distinguish between the different subtypes; and (iii) in situ hybridization is one of the most convincing methods to identify the cytokine-expressing cells because it is based on messenger RNA (mRNA) detection of the targeted genes [47].

In the present study, the cryosections of non-decalcified rat femur surrounded by muscle were investigated by immunostaining, in situ hybridization, and in situ HCR to identify phenotypes of macrophages involved in bone regeneration. In these challenging conditions, expression of CD68, CD163, IL-1β, IL-6 and β-actin genes was successfully detected resulting for the first time in the identification and localization of M2b-like macrophages in vivo in the bone of rats during bone regeneration.

## 2. Materials and Methods

### 2.1. Rat Animal Model

All experiments were approved by the IRBA Institutional Animal Care and Use Committee (protocol 65 DEF_IGSSA_SP). Surgeries were carried out in an accredited animal facility. Eight-week-old (200 g average weight) male Sprague Dawley rats (Charles River Laboratories, Freiburg, Germany) were housed individually in cages in a temperature and light-controlled environment with food and provided water ad libitum. Before collecting femurs with muscles, animals were euthanized at 12 weeks old with an overdose of sodium pentobarbital (150 mg/kg) administrated intraperitoneally.

### 2.2. Embedding and Cryosectioning of the Entire Femur (Bone and Muscle Together) of the Rat 

Embedding and cryosectioning methods were performed as described in [49]. RNase-free instruments, materials, and buffers were used to collect bone samples. After euthanasia of the rat, the whole femur was cleaned rapidly, and a part of the muscles around the bone was kept. The femur was placed at the bottom of the embedding mold and covered with cryomount medium (CM) (00890-EX, HistoLab, Askim, Norway). Samples were snap-frozen with 2-methyl butane cooled in liquid nitrogen to obtain a block.

### 2.3. Histological Staining

Histological staining was performed as described in [49]. Hematoxylin and phloxin (HP) staining was performed as follows: the sections were incubated in several successive baths: 40 s in a hemalum (11,487, Merck, Darmstadt, Germany) buffer (0.2 g hemalum, 5 g aluminum potassium sulfate in 100 mL distilled water), 3 min in water, 30 s in a phloxin (15,926, Merck, Darmstadt, Germany) buffer (0.5 g phloxin in 100 mL distilled water), 1 min in water, 2 min in 70% ethanol, 30 s in 95% ethanol, 1 min in 100% ethanol, and 1 min in 100% ethanol. In the end, nuclei were colored in blue and cytoplasm in pink.

### 2.4. Immunofluorescence

Sections of the rat femur were fixed in 4% (*w*/*v*) paraformaldehyde (PFA (P6148, Sigma, Lezennes, France) in PBS (phosphate-buffered saline without Ca and Mg, GAUPBS0001, Eurobio, Les Ulis, France). After three washes in PBS, the sections were permeabilized for 15 min with 0.5% (*v*/*v*) Triton X100 buffered with PBS. The non-specific binding sites were blocked with Emerald Antibody Diluent (936B-08, Sigma, Lezennes, France) for 1 h at room temperature. Then, sections were incubated overnight at 4 °C with the primary rabbit anti-CD68 (ab125212, Abcam, Amsterdam, The Netherlands) antibody at 1:1000 di lution; the primary goat anti-CD206 (C20) (sc-34577, Santa Cruz Bio., Heidelberg, Germany) antibody at 1:1000 dilution; the primary rabbit anti-CD163 (ab182422, Abcam, Amsterdam, The Netherlands) antibody at 1:500 dilution; and the primary rabbit anti-Iba1 (ab178846, Abcam, Amsterdam, The Netherlands) antibody at 1:200 dilution. Then, the sections were washed in PBS and incubated with the secondary anti-rabbit Alexa Fluor 488 (A-21206, Thermo Scientific, Waltham, MA, USA) antibody at 1:500 dilution and the secondary anti-goat Alexa Fluor 568 (ab175704, Abcam, Amsterdam, The Netherlands) antibody at 1:500 dilution for 2 h at room temperature. Finally, sections were washed in PBS for 20 min and mounted using a Fluoroshield mounting medium with DAPI (ab104139, Abcam, Amsterdam, The Netherlands). The fluorescence was detected using an epifluorescence microscope DM6000 (Leica, Schönwaldeglien, Germany) equipped with monochrome and color digital cameras.

### 2.5. RT-qPCR

Frozen femur samples were homogenized in liquid nitrogen. Total RNA was then isolated using an RNeasy Fibrous Tissue mini kit (HB-0485, Qiagen, Courtaboeuf, France) according to the m anufacturer’s recommendations. RNA extracts were recovered with 20 µL RNase-free water. Reverse transcription (RT) was performed with oligo-dT primers following the instructions of the Sensiscript transcription kit (205211, Qiagen, Courtaboeuf, France); cDNA synthesis was carried out for 1 h at 37 °C using 50 ng of RNA with 10 µM oligod(T) primers, RNase inhibitor (2 IU), and Sensiscript reverse transcriptase.

Real-time qPCR was carried out in a 20 µL final volume using LC480 SybrGreen I Mastermix (Roche Applied Science, Mannheim, Germany) using 0.25 µL of cDNA. Oligos designed for RT-qPCR experiments are listed in Table S1.

### 2.6. In Situ DIG Hybridization

In situ hybridization methods were performed as described in [49]. Oligos designed for in situ hybridization experiments are listed in Table S2.

Digoxigenin (DIG)-labeled cRNA probes were used for in situ hybridization. Briefly, the tissues were fixed in 4% (*w*/*v*) in PBS/paraformaldehyde (PBS, *w*/*o* Ca and Mg, GAUPBS0001, Eurobio, Les Ulis, France; PFA, P6148, Sigma, Lezennes, France) for 30 min and then treated with 100% methanol for 15 min and air-dried. The sections were pre-hybridized for 2 h at 45 °C in a pre-hybridization buffer (50% formamide (GHYFOR0402, Eurobio, Les Ulis, France), 0.5× sodium ch loride citrate (SSC) (GHYSSC007, Eurobio, Les Ulis, France) buffer, 50 µg mL−1 heparin (H3393, Sigma, Lezennes, France), 100 µg mL^−1^ transfer RNA, and 0.1% (*v*/*v*) Tween 20 (822184, Merck, Darmstadt, Germany). Finally, the sections were incubated overnight at 45 °C with the RNA probes (2 µL probe in 200 µL hybridization buffer (50% formamide, 100 µg mL^−1^ transfer RNA, 7.5% (*v*/*v*) Tween 20, 8.5% NaCl, 20% dextran sulfate (GHYDEX000T, Eurobio, Les Ulis, France), and 2.5× Denhardt’s Solution (50× stock, D2532, Sigma, Lezennes, France)), which were previously denaturized for 2 min at 80 °C in the hybridization buffer. Non-specific hybrids were dissociated with the following washes: 30 min in 0.1× SSC + 0.5% SDS at 45 °C, 2 h in 2× SSC + 50% formamide at 45 °C, 5 min in NTE (0.5 M NaCl, 10 mM Tris pH 8, 1 mM EDTA) at 45 °C, 30 min in NTE + 10 mg mL^−1^ Rnase A (10109169001, Roche, Boulogne-Billancourt, France) at 37 °C, 1 h in 2× SSC + 50% formamide at 45 °C, 2 min in 0.1× SSC at 45 °C, and finally 15 min in PBS at RT.

Immunodetection of the DIG-labeled probes was performed using an anti-DIG antibody coupled to alkaline phosphatase as described by the manufacturer (11093274910, Roche, Boulogne-Billancourt, France). Afterward, the sections were incubated for 1–2 days in a buffer containing 337 µL BCIP (5-Bromo-4-chloro-3-indolyl-phosphate) and 225 µL NBT (Nitroblue tetrazolium chloride) in a 50 mL solution (100 mM Tris pH 9.5, 100 mM NaCl and 50 mM MgCl_2_) until a blue precipitate adhering to the sections were formed. The DIG sections were observed with an epifluorescence microscope DM6000 (Leica, Schönwaldeglien, Germany) equipped with monochrome and color digital cameras, while the HCR sections were observed with a confocal microscope (LSM700, Zeiss, Dresden, Germany).

### 2.7. In Situ HCR Hybridization

In situ hybridization methods were performed as described in [49]. Oligos designed for in situ hybridization experiments are listed in Table S2.

The HCR protocol of Choi and colleagues (2014, 2016, 2018, 2020) was performed with some modifications as described below to enhance mRNA localization in the femur of the rat [50,51,52,53].

The sections were pre-hybridized for 10 min at RT in a hybridization buffer (50% formamide, 5× SSC, 9 mM citric acid pH 6, 50 µg mL^−1^ heparin, 1× Denhardt’s Solution, 0.1% (*v*/*v*) Tween 20 and 10% dextran-sulfate). Previously, the hybridization probes (2 pmol per slide) were denatured for 2 min at 80 °C. Finally, the sections were incubated in a hybridization buffer together with probes overnight at 45 °C. Nonspecific hybrids were dissociated with the following washes: 30 min in 0.1× SSC + 0.5% SDS at 45 °C, followed by 2 h in 2× SSC + 50% formamide at 45 °C, then 2 min in 0.1× SSC at 45 °C, and finally 15 min in PBS at RT.

Sections were first incubated for 2 h at RT with an amplification buffer (5× SSC, 0.1% (*v*/*v*) Tween 20, 10% dextran-sulfate, and 100 µg mL^−1^ salmon sperm ADN) and subsequently for 12 to 16 h with the DNA hairpins marked with a fluorophore (Alexa Fluor488) (diluted in amplification buffer as previously described). The hairpins were previously heated at 95 °C for 90 s and cooled to RT for 30 min. The sections were then washed 2× 30 min in 5× SSCT (5× SSC and 1% (*v*/*v*) Tween 20) and 5 min with 5× SSC without Tween at RT.

### 2.8. Microspectroscopical Analysis

For spectroscopic analysis, an LSM 780 (Carl Zeiss, Jena, Germany) confocal microscope was used to acquire a lambda stack of 10 nm wavebands between 425 nm and 625 nm. Then, Zen Black software (Carl Zeiss, Jena, Germany) built-in plugin was used to perform linear unmixing using the automatic component extraction algorithm. It was possible to extract Alexa 488 spectrum, and the second spectrum was considered the sum of all other sources of endogenous fluorescence (autofluorescence).

### 2.9. Quantification of HIS by ImageJ

To quantify the percentage of the marked region on in situ hybridization slides, each slide was acquired with the Hamamatsu Nanozoomer S60 at 20x (Tokyo, Japan). On the virtual slide, three regions of interest (ROI) were drawn in the immediate neighborhood of the biomaterial; three others were done away from it (internal negative control). Each zone was analyzed using the Fiji software. The ratio between the surface of the marked region and the surface of the entire ROI was computed after color deconvolution and thresholding (using the MaxEntropy algorithm).

Statistical analyses were performed with a *t*-test.

## 3. Results

### 3.1. Detection of Immune Cell Accumulation around Biomaterial

**Figure 2:**
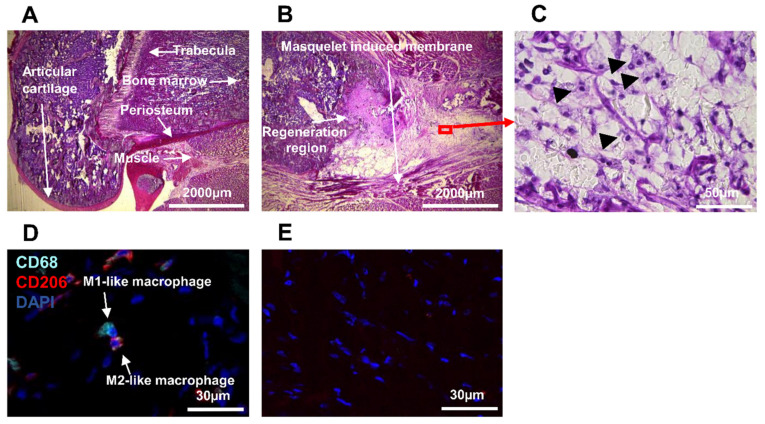
Histologic and microscopic analysis of rat femur: (**A**–**C**) histologic analysis of the femurs; histopathological image of (**A**) non-operated; and (**B**) operated femurs of rats stained with hematoxylin and phloxin; (**C**) expanded view: high magnification image of the area within the red rectangle in image (**B**); (**D**,**E**) identification of macrophages M1 and M2 in the operated femur; anti-CD68 (Alexa488, turquoise fluorescence), labeling the M1 and M2 macrophages; anti-CD206 (Alexa568, red fluorescence) labeling the M2 macrophages and satellite cells; nuclear staining with DAPI (blue fluorescence).

It was shown that biomaterial triggered the recruitment of macrophages at the site of the bone wound and enhanced wound healing [1,7,32,54]. Rat femur was operated on with biomaterial (with the Masquelet induced membrane technique), and three weeks later the whole bone together with femur and biomaterial was harvested. The non-operated femur from the second leg of this rat was used as a control (called non-operated femur). The Masquelet induced membrane technique consists of two different operative phases. In the first operation, a fixator was placed around the bone, and the missing bone fragments were filled with biomaterial. Three weeks after surgery, a Masquelet induced membrane was formed around the biomaterial. Three weeks later, during the second operation, the biomaterial was removed, but the Masquelet induced membrane remained, and the biomaterial was replaced with a bone graft. To examine the architecture and the regeneration region of the bone, cryosections of femurs were analyzed by Hematoxylin and phloxin (HP) histological coloration (Figure 2). The control femur was used as a reference to observe bone and muscle in their native structure (Figure 2A). In the operated femur, the Masquelet induced membrane, the regenerated region close to the Masquelet induced membrane, and the biomaterial were observed (Figure 2B). Some immune cells were identified by their structure at higher microscope magnification in the regeneration region (Figure 2C), hypothesized as macrophages.

In situ visualization of macrophages is quite problematic. The cluster of differentiation (CD) CD68 protein is one of the most common monocytes/macrophages marker proteins [55], but in other mononuclear phagocyte cells and non-hematopoietic cells (mesenchymal stem cells, fibroblast, endothelial, and tumor cells) weak expression can be detected [56]. Other markers such as CD206 and CD163 mostly recognize M2 macrophages, but CD206 protein is also expressed in satellite and CD163 protein in dendritic cells [56,57]. In situ identification of human and mouse M2 macrophages can be performed by double immunolabeling with CD206 or CD163 together with CD68 antibodies (Table 1) [47,58].

The same region was examined by the traditional immunostaining method (with anti-CD68 and anti-CD206 antibodies) to detect if M1-like or M2-like macrophages were localized in the regeneration region (Table 1). We observed M1-like macrophages expressing CD68 protein only and M2-like macrophages expressing both CD68 and CD206 proteins (Figure 2D). In the absence of a primary antibody (negative control), no expression of these markers was detected (Figure 2E).

**Figure 3:**
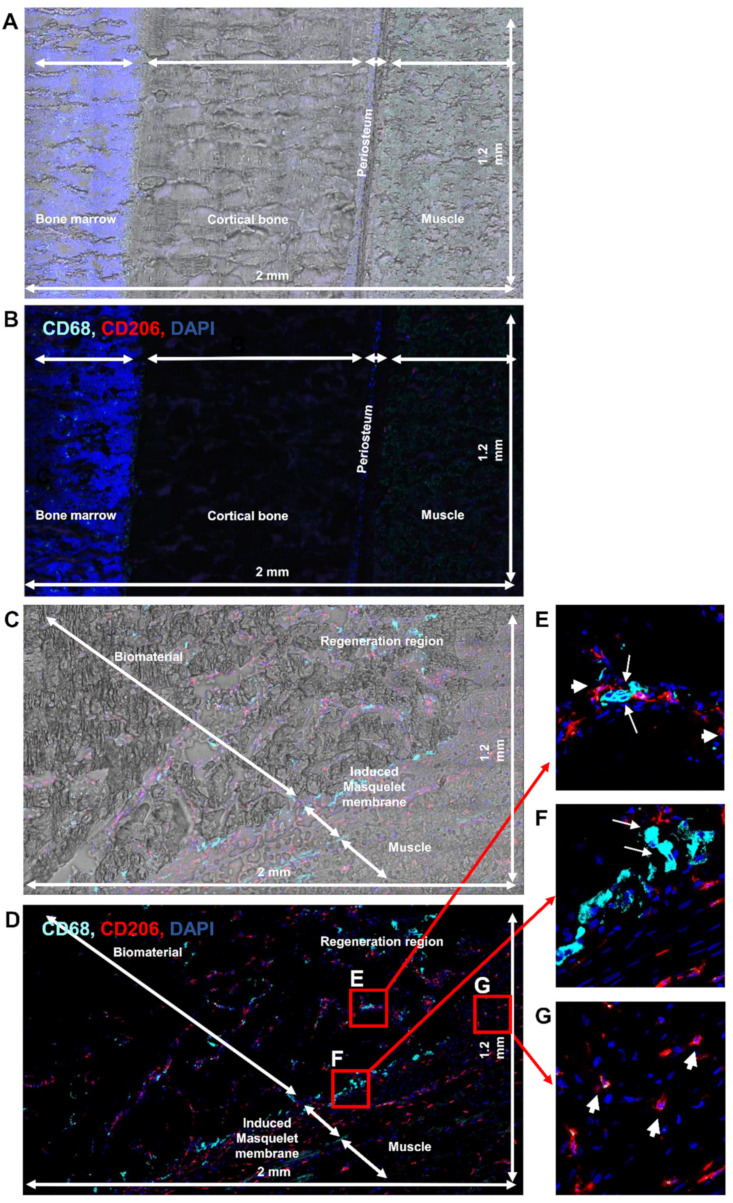
Identification of M1-like and M2-like macrophages in the rat femurs: (**A,B**) immunolabeling with anti-CD68 and CD206 antibodies of the non-operated; and (**C**–**G**) operated femurs; bright-field image together with immunolabeling of the non-operated (**A**) and operated (**C**) femurs; (**E**–**G**) expanded view: high magnification image of the area within the red rectangle in image C; anti-CD68 (Alexa488, turquoise fluorescence), labeling the M1 and M2 macrophages; anti-CD206 (Alexa568, red fluorescence) labeling the M2 macrophages and satellite cells; nuclear staining with DAPI (blue fluorescence); thin arrow: M1-like macrophages; thick arrow: M2-like macrophages.

To go further in our investigation, immunostaining was performed on both the non-operated femur and the operated femur (Figure 3) in large sections (2 mm × 1.2 mm). Using bright field microscopy, the non-operated femur showed a representative architecture of the bone (Figure 3A) and an absence of fluorescence signal (Figure 3B). In the operated femur, the three expected regions (the regeneration region, the Masquelet induced membrane, and surrounding muscle) were observed (Figure 3C). Using CD68 and CD206 immunostaining, both M1-like and M2-like macrophages were detected (Figure 3D). However, the M1-like and M2-like macrophage repartition differed among the three zones (Figure 3E–G). A similar amount of M1-like and M2-like macrophages were observed in the regenerating region (Figure 3E) compared to the interface (between the regeneration region and Masquelet induced membrane) region, where mostly M1-like macrophages (Figure 3F) and a predominantly M2-like macrophage population were detected (Figure 3G).

Another widely accepted macrophage marker is the ionized calcium-binding adaptor molecule 1 (Iba1) (Table 1) [59,60,61]. Labeling with the anti-Iba1 antibody together with the anti-CD206 antibody showed very similar results (Figure S1). The only difference that has been detected is that fewer M1-like macrophages have been detected by Iba1 labeling in the interface region than with CD68 labeling. This result suggests that single CD68 positive cells include other cells than macrophages that have not yet been identified.

**Figure 4:**
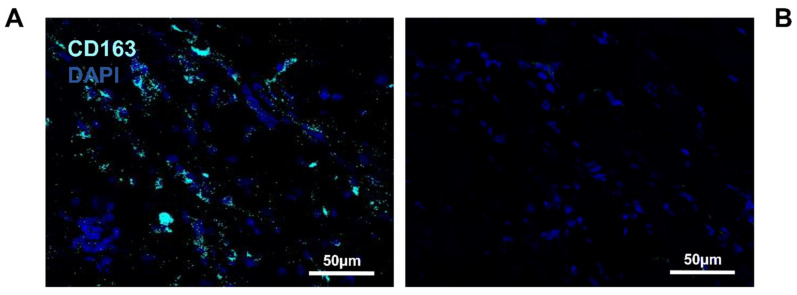
Identification of M2-like macrophages in the operated femur: (**A**) immunolabeling of the operated femur with anti-CD163; (**B**) negative control of the immunolabeling; anti-CD163 (Alexa488, turquoise fluorescence), labeling the M2 macrophages; nuclear staining with DAPI (blue fluorescence).

In summary, both M1-like and M2-like macrophages were identified in the injured region of the operated femur. After quantification of 1000 cells, 16.55% (CD68/CD206) and 17.5% (Iba1/CD206) were identified as M2-like macrophages compared to 6.17% (CD68/CD206) and 4.1% (Iba1/CD206) of M1-like macrophages (Table 2). To confirm this result, immunostaining with an anti-CD163 antibody was performed because this protein is also expressed by M2-like macrophages [16]. Next, the CD163 and CD206 co-labeling was performed to identify the M2-like macrophages and satellite cells in the same pictures (Figure S2). In this condition, 21.6% of M2-like macrophages were identified (Figure 4A and Table 1), while no expression of the protein was detected in the negative control (Figure 4B).

### 3.2. Detection of Macrophages in Other Regions

**Figure 5:**
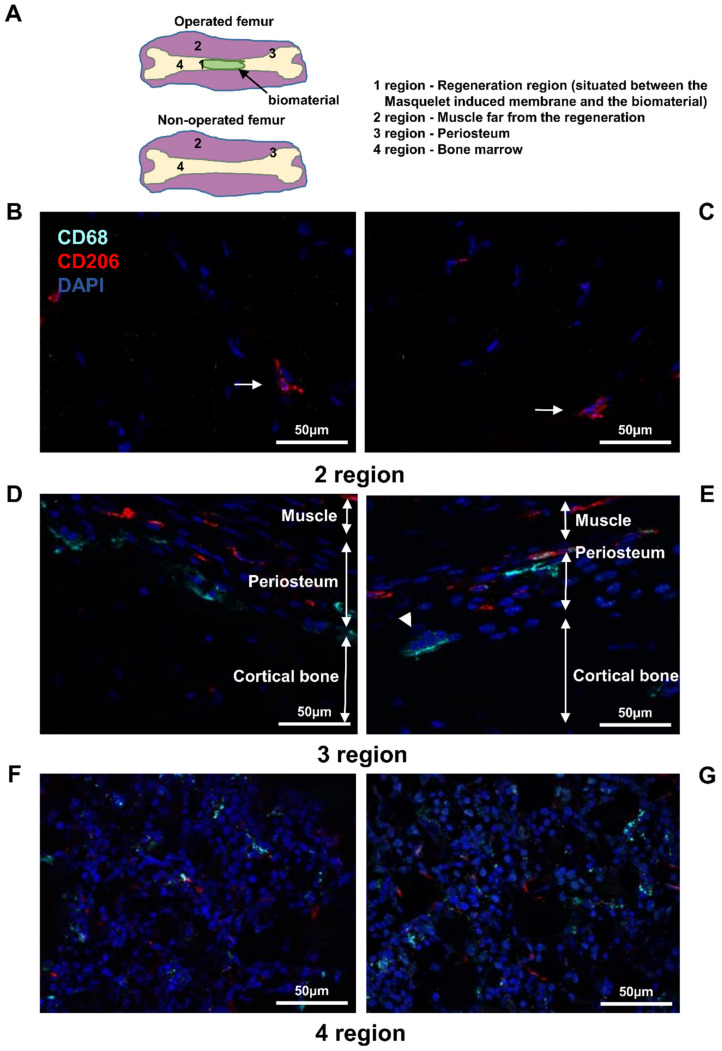
Identification of M1-like and M2-like macrophages in the rat femurs: (**A**) presentation of the different regions of the femurs; (**B**,**D**,**F**) operated; and (**C**,**E**,**G**) non-operated femurs labeled with anti-CD68 and anti-CD206 antibodies; anti-CD68 (Alexa488, turquoise fluorescence), labeling the M1 and M2 macrophages; anti-CD206 (Alexa568, red fluorescence) labeling the M2 macrophages and satellite cells; nuclear staining with DAPI (blue fluorescence); thin arrow: satellite cells; arrowhead: osteoclast.

Previously we showed the capacity to detect macrophages using immunostaining in a non-decalcified cryo-fixed bone in the regeneration region (region 1). We further examined whether the different regions of the femur far from the injury region contained macrophages with phenotypic differences. In this context, three other regions were investigated: the muscle far from the bone or the wound (region 2), the periosteum (region 3), and the bone (region 4) (Figure 5 and Figure S3). No difference between operated and non-operated femur tissues were observed (Figure 5). In region 2, no macrophages were present; only satellite cells could be detected (Figure 5B,C). In region 3, both M1-like and M2-like macrophages were detected close to osteoclasts, as it is multinucleated cells expressing CD68 (Figure 5D,E). In region 4, several cells expressing CD68 protein were detected. This result seemed to be coherent as macrophages were also localized in the bone marrow. Surprisingly, cells expressing only the CD206 protein were also detected (Figure 5F,G). This result requires further examination. No expression of both CD68 and CD206 markers was detected in the negative control (Figure S3).

### 3.3. Detection of Macrophages Based on Their Autofluorescence Feature

**Figure 6:**
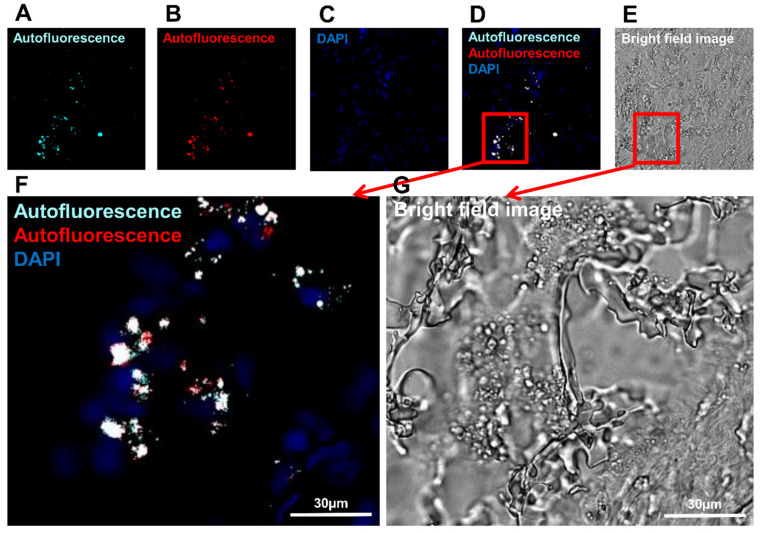
Observation of the autofluorescence in the operated rat femur: (**A**) detected autofluorescence after excitation with light (488 wavelengths, turquoise fluorescence); (**B**) detected autofluorescence after excitation with light (568 wavelengths, red fluorescence); (**C**) nuclear staining with DAPI (blue fluorescence); (**D**) merged image; (**E**) bright-field image; (**F**) expanded view: high magnification image of the area within the red rectangle in image (**D**); (**G**) expanded view: high magnification image of the area within the red rectangle in image (**E**).

It is well known that bone tissue has strong autofluorescence [62,63]. This makes it rather difficult to analyze the operated tissue with immunolabeling (using fluorophore-labeled antibody) because there was an abundant autofluorescence in the regenerating region (Figure 6). Autofluorescence may appear from structural proteins, such as collagen and elastin, but other endogenous fluorophores, such as Flavin-type molecules, are also often localized in cells [64,65]. In particular, immune cells, such as macrophages and granulocytes contain a large amount of phagosome/phagolysosome in their cytosol [66]. Sections of the operated femur tissues had relatively intense (excitation with 488 nm (Figure 6A–D,F) and excitation with 568 nm (Figure 6B–D,F)) autofluorescences exclusively in the cells with endosome-like structure (Figure 6E,G). The sections of the operated femur were subjected to different treatments, TrueVIEW Autofluorescence Quenching Kit, Blue Evans, or Black Soudan, to get rid of endogenous fluorescence (data not shown). Unfortunately, the endogen fluorescence was resistant to all treatments used so far. s

### 3.4. Investigation of Macrophage Fluorescence by Microspectroscopic Analysis

**Figure 7:**
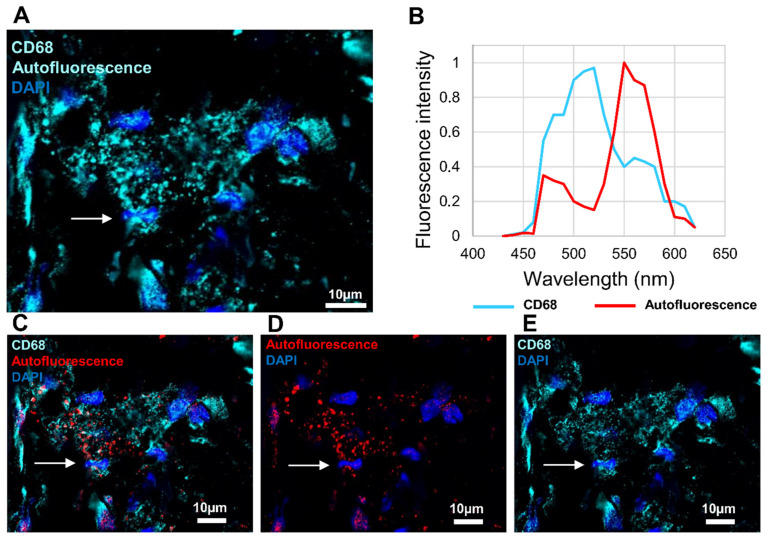
Identification of the in situ autofluorescence by microspectroscopy analysis. In the operated femur, macrophages were labeled with anti-CD68 antibody. Tissues were excited at 488 nm. (**A**) Emission of the autofluorescence and the antigen-specific fluorescence (Alexa Fluor 488) with turquoise fluorescence. (**B**) Microspectroscopycal analysis of Alexa Fluor 488 emission (turquoise line) and autofluorescence (red line). (**C**) Separation of autofluorescence emission (red fluorescence) and antigen-specific fluorescence (Alexa Fluor 488) (turquoise fluorescence). (**D**) Emission of the autofluorescence (red fluorescence). (**E**) Emission of the antigen-specific fluorescence (Alexa Fluor 488) (turquoise fluorescence). Nuclear staining with DAPI (blue fluorescence). Fine arrow: macrophages.

As the endogen fluorescence was very strong in the bone tissue, further investigation was needed for macrophage characterization. Turquoise fluorescence was detected in macrophages, but the autofluorescence prevented any conclusion as both specific fluorescence (coming from the immune signal) and autofluorescence were detected in a mixed signal (Figure 7A). Microspectroscopical analysis in situ facilitates the differentiation between specific fluorescence and autofluorescence. The ratio between the intensity of specific fluorescence and autofluorescence should be higher than three to conclude that the autofluorescence is not disturbing. This technique was applied to excited signals at 488 nm to separate them from their characteristic emission spectra. The emission spectrum of specific fluorescence reached a maximum at 520 nm, whereas the emission spectrum of autofluorescence peaked at approximately 550 nm (Figure 7B). The intensity of the specific CD68 fluorescence was approximately the same as the intensity of the autofluorescence (Figure 7B). Using this technique, two subtracted images were extracted from Figure 7C: the autofluorescence signal (Figure 7D) and the specific CD68 fluorescence of the macrophages (Figure 7E). In the resulting image, we can distinguish autofluorescence from the CD68 specific signal in cells (Figure 7E).

### 3.5. Detection of IL-1β and IL-6 Cytokines in the Operated Femur

**Figure 8:**
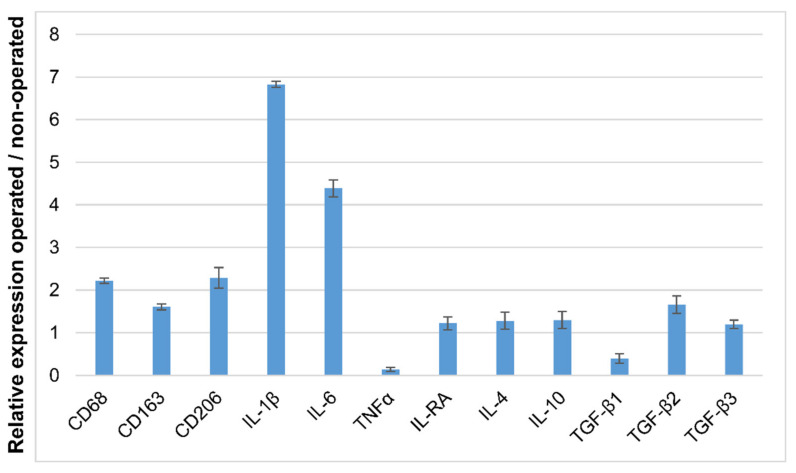
Expression of the different marker genes and cytokines in the operated femur; qRT-PCR analysis of the different gene expressions. This result is the average of three measurements.

The presence of M1-like and mostly M2-like macrophages was shown earlier in the wounded bone tissue with immunofluorescence techniques. The next step was to determine the M2-like macrophages based on their cytokine expressions. Each subtype of M2-like macrophages (M2a, M2b, M2c, or M2d) secretes a different panel of cytokines. The next step was to identify the types of cytokines produced in the operated femur using RT-qPCR. All three markers showed higher expression in the operated femur (2.2 times more CD68, 1.61 times more CD163, and 2.29 times more CD206 mRNA) (Figure 8). Among the tested genes, two cytokines showed much higher expression, IL-1β (6.89 times more) and IL-6 (4.44 times more), in the operated femur compared to the non-operated femur (Figure 8). The only M2 macrophage that produces these two cytokines is the M2b macrophage (Figure 1). The in situ DIG technique is a commonly used, non-radioactive IHS method because it is very sensitive, but it has the limitation that it only allows analysis of a single gene in a sample.

### 3.6. Identification of Macrophage Phenotype Using Both I DIG and In Situ HCR Techniques

In situ HCR was chosen to further characterize the macrophages. The advantage of this technique is that it is more sensitive than FISH, and unlike in situ DIG, it allows the identification of several genes at once. Our next question was whether we could detect M2b-like macrophages in situ using this technique in the operated rat bone. Since cytokines are usually secreted [48,67], immunostaining is not suitable to distinguish between different subtypes. Indeed, it is quite challenging to identify which cells produce the secreted protein. For this reason, an in situ hybridization technique was used to reveal the subtypes of M2-like macrophages as it allows the detection of the mRNA coding for those cytokines.

**Figure 9:**
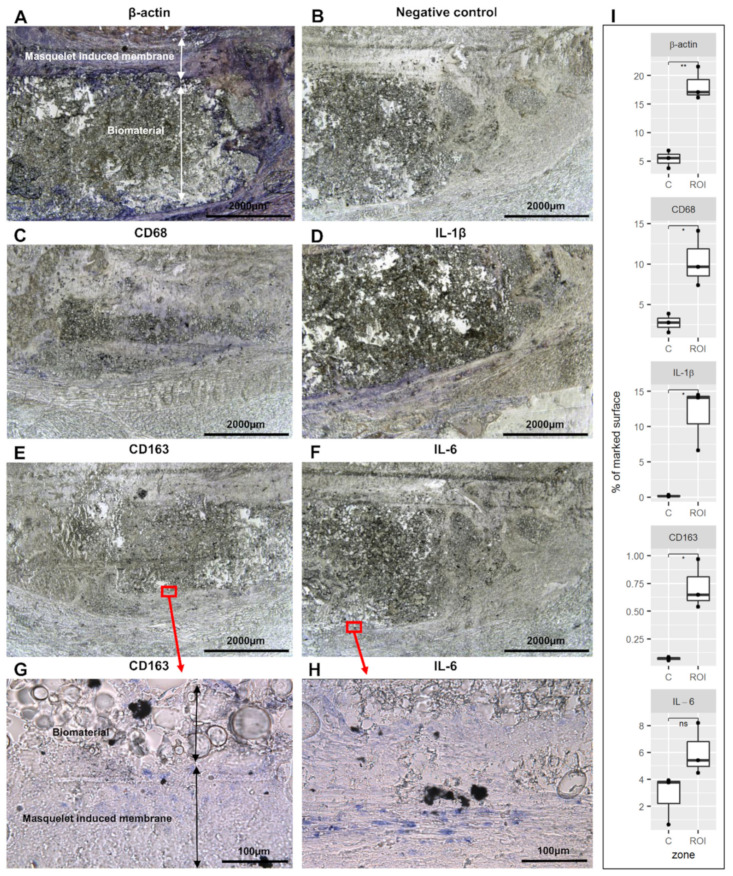
In situ hybridization in the operated rat femur: (**A**) expression of β-actin mRNA (positive control); (**B**) negative control; (**C**) CD68 mRNA; (**D**) IL-1β mRNA; (**E**) CD163 mRNA; (**F**) IL-6 mRNA; (**G**) expanded view: high magnification image of the area within the red rectangle in image (**E**); (**H**) expanded view: high magnification image of the area within the red rectangle in image (**F**); (**I**) the stained area of the picture was quantitatively analyzed using ImageJ. The Masquelet induced membrane region was compared with the control region (**C**) (n = 3). ** *p* < 0.01, * *p* < 0.5, ns = not significant, compared to control region.

For the first time, in situ hybridization with a digoxigenin-labeled cRNA probe (in situ DIG) was used because the labeling remains stable, and it is an advantage to examine the labeling architecture. This technique is the most widely used non-radioactive ISH method because it is very sensitive, unfortunately, only one mRNA per section can be detected [68]. The expression of β-actin, IL-1β, IL-6, CD68, and CD163 genes was identified (Figure 9). The β-actin was used as a positive control (Figure 9A). In the absence of a specific probe, no expression was observed (Figure 9B). The mRNA expression of CD68, CD163, IL-1β, and IL-6 genes (Figure 9C–F) was detected around the biomaterial. Both CD68 (Figure 9C) and IL-1β mRNA expression (Figure 9D) was very intense. The CD163 (Figure 9E) and IL-6 (Figure 9F) mRNA expressions were much weaker, but a higher magnification showed a clear labeling (Figure 9G,H). In addition, β-actin, CD68, CD163, IL-1β, and IL-6 signals were quantified in the operated femur. Upregulation of these genes was detected in the Masquelet induced membrane region.

The same labeling was performed on the non-operated femur sections. In the non-operated section, abundant expression of the β-actin mRNA was detected (Figure S4A), and in the negative control (Figure S4B), expression was not observed. The CD68 (Figure S4C) and IL-1β mRNA (Figure S4D) expressions were intense in the bone marrow, but no expression in the other part of the femur was detected. No expression was found with a small magnification of the CD163 (Figure S4E) and IL-6 (Figure S4F) mRNA. The CD163 expression was detectable only with higher magnification in the periosteum (Figure S4G). IL-6 mRNA was present in the bone marrow (Figure S4H).

**Figure 10:**
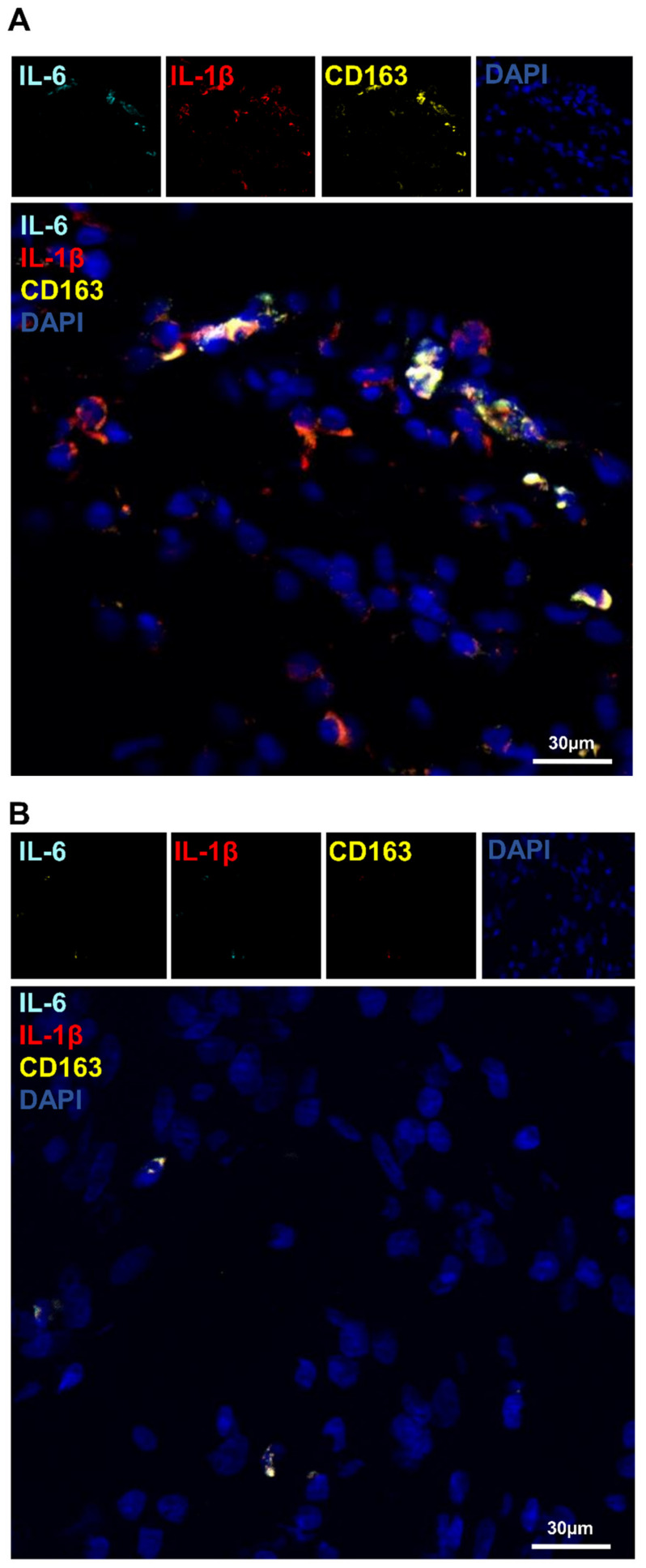
Identification of the M2b-like macrophages in the operated femur by in situ hybridization combined with hybridization-chain-reaction detection (in situ HCR): (**A**) in situ hybridization combined with hybridization-chain-reaction detection (in situ HCR); (**B**) negative control: Probe-IL-6 (Alexa488, turquoise fluorescence), Probe-IL-1β (Alexa546, red fluorescence), Probe-CD163 (Alexa647, yellow fluorescence), nuclear staining with DAPI (blue fluorescence).

Because co-labeling was not possible with the in situ DIG technique, in situ hybridization coupled with hybridization-chain-reaction detection (in situ HCR) was performed on cryosections of the bone. Indeed, three different mRNA expressions were co-detected, taking into account the rapid loss of signal of fluorophores within two weeks after labeling. Using the in situ DIG method, a high expression of IL-1β and IL-6 was detected in the regenerating bone. To examine whether M2-like macrophages were expressing these two cytokines, in situ HCR was performed with CD163, IL-1β, and IL-6 probes altogether (Figure 10A). Macrophages co-expressing the three signals were identified in the regenerated region of the operated femur, while no expression was detected in the negative control (Figure 10B). By random examination of the fluorescence of 100 cells labeled with a CD163 probe, the percentage of the M2b-like macrophages was approximately 68% of the M2-like macrophages.

## 4. Discussion

Bone and muscle tissues constantly interact with each other by secreting cytokines and other soluble factors [69]. Analysis of cytokine expressions during bone regeneration is very important for developing new therapeutic approaches. We aimed to analyze the different macrophage-specific cytokine expressions during bone regeneration in the sections of the entire femur surrounded by muscle. Before sectioning, the tissues must be fixed and embedded in paraffin. In our case, paraffin embedding was not possible because the muscle significantly slowed down (around 5 weeks) the decalcifying solution (ethylene diamine tetra acetic acid (EDTA)) from penetration and did not allow the use of ISH techniques in these sections (data not shown) due to the mRNA degradation. To avoid this problem, an improved version of the CryoJane tape transfer system was developed in our laboratory to obtain the cryosections of the entire femur surrounded by muscle, which was suitable for ISH techniques [49].

The role of macrophages in tissue development, homeostasis, and wound healing is essential [17,19,21,22,37,38,39]. Bone regeneration is a very complex process. The continuous interaction between bone-forming cells and macrophages is essential for successful bone healing [1,70,71]. However, their role in bone regeneration remains poorly understood. Subsequently, a deeper understanding of their involvement after an injury is essential to developing new therapeutic strategies for bone repair. A broad spectrum of biomaterials, differing in their structure, porosity, composition, and chemistry, is used as therapeutic strategies for tissue repair. It is known from in vitro studies that macrophage polarization remains biomaterial dependent. Data in the literature suggest that the host response in the in vivo condition is more complex which limits the interpretation of in vivo results compared to in vitro observations [72,73,74]. Previously, it was speculated that M1 and M2 macrophages subsequently participate in a different stage of bone regeneration [1,75,76,77]. Three weeks after lesion/surgery, both M1-like and M2-like types of macrophages were detected in the injured bone tissue. Mostly M2-like macrophages were identified, while few but detectable M1-like macrophages were observed. Our results suggest that bone regeneration is a complex process, and in contrast to our expectation, it requires the continuous presence of two types of macrophages. Several publications suggested that macrophages exert their effects on bone regeneration through secreted cytokines [1,8,36,38]. Vi and collaborators (2015) demonstrated that even the conditioned medium of the macrophages was able to activate the “mineral deposition and bone formations” [8].

Macrophage subtypes produce diverse cytokines at different stages of regeneration. Identification of cytokine-expressing cells by immunolabeling is quite complicated because these proteins and peptides are secreted, making it very difficult to determine which cells have produced them. In this work, we demonstrated the possibility to identify macrophages in bone after the detection of cytokines expressed by macrophages with in situ hybridization coupled with in situ HCR detection methods. This tool allows in situ characterization of macrophages during bone regeneration, which was not possible earlier. Fluorescence in situ hybridization (FISH) appeared in the 1980s [78,79,80]. The advantage of this method compared with in situ DIG was that the expression of several mRNAs could be analyzed in the same section [47,81]. However, it was only suitable for detecting highly expressing mRNA. Unfortunately, the expression of cytokines is usually low. The in situ HCR [49,50,51,52,53] method is a promising new method to study gene expression in bone tissues. This technique allows the detection of multiple mRNAs also in the same section and is suitable for the visualization of mRNA with low expression.

Other groups exhibited that at the bone damage location, M1 macrophages secreted pro-inflammatory cytokines (TNF, IL-1β, and IL6) and induced the recruitment of the mesenchymal stem cells (MSC) and other progenitor cells at the local site. Cytokine expression was investigated in the operated and non-operated tissues by qPCR and in situ DIG methods. Interestingly, very high expression of IL-6 and IL-1β was detected by qPCR, and in situ DIG showed that these two cytokines were localized around the biomaterial. M1-like and M2-like macrophages were present in regenerating bone although the M2-like macrophages were more abundant. The M2b macrophages are the only M2-like macrophages that co-secrete these two cytokines. We found that the in situ HCR method is a good compromise in macrophage identification and allows in situ determination of the presence of the M2b-like macrophages.

## 5. Conclusions

In summary, at least five observations were highlighted: (1) There was a significant difference between non-operated and operated bone tissues. With immunostaining, in the operated bone tissue we observed mostly M2-like macrophages with smaller quantities of M1-like macrophages. No macrophages were found in the non-operated bone tissue. (2) The localization of the macrophages was limited to the wounded area. (3) The operated tissue showed a strong IL-1β and IL-6 cytokine expression as measured by RT-qPCR detection. (4) In addition, the in situ HCR method has been proved useful as it allows selectively exploring the RNA expressions of the cytokines and macrophage markers in the cells of the wounded bone tissue. With this technique, it was possible to identify the in vivo M2b-like macrophages in the wounded bone tissue. (5) It was pointed out that microspectroscopical analysis is very important in macrophage characterization because it can differentiate between fluorescence and autofluorescence.

Finally, this work highlighted the limits of immunostaining and the interest in in situ HCR method providing valuable information for in vivo macrophage characterization. This technique is an important approach for analyzing the biological mechanism of bone regeneration and offers a new perspective in the field of regenerative medicine.

## Figures and Tables

**Table 1:** Cell phenotypes.

	CD68	CD206	CD163	Iba1
M1-like macrophages	+	−	−	+
M2-like macrophages	+	+	+	+
Satellite cells	−	+	−	−

**Table 2:** Quantification of M1-like and M2-like macrophages in the regeneration region of the operated femur (quantitative analysis, based on random examination of 1000 cells in each of the conditions).

	M1-like Macrophages	M2-like Macrophages
CD68/CD206	6.2%	16.6%
Iba1/CD206	4.1%	17.5%
CD163	/	21.6%

## Data Availability

Not applicable.

## Supplementary Materials

The following supporting information can be downloaded at: https://www.mdpi.com/article/10.3390/biomedicines10061385/s1. Figure S1: Identification of M1-like and M2-like macrophages in the rat femurs; (A–D) immunolabeling with anti-Iba1 and CD206 antibodies of the operated femurs; (B–D) expanded view: high magnification image of the area within the red rectangle in image A; anti-Iba1 (Alexa488, turquoise fluorescence), labeling the M1 and M2 macrophages; anti-CD206 (Alexa568, red fluorescence) labeling the M2 macrophages and satellite cells; nuclear staining with DAPI (blue fluorescence); thin arrow: M1-like macrophages; thick arrow: M2-like macrophages. Figure S2: Identification of M2-like macrophages and satellite cells in the rat femurs: (A) immunolabeling with anti-CD163 and CD206 antibodies of the operated femurs; (B) negative control; anti-CD163 (Alexa488, turquoise fluorescence), labeling the M2-like macrophages; anti-CD206 (Alexa568, red fluorescence) labeling the M2-like macrophages and satellite cells; nuclear staining with DAPI (blue fluorescence); thin arrow: M2-like macrophages; thick arrow: satellite cells. Figure S3: Identification of macrophages M1-like and M2-like in the femurs of the rat; (A) negative control of Figure 4B; (B) negative control of Figure 4C; (C) negative control of Figure 4D; (D) negative control of Figure 4E; (E) negative control of Figure 4F; (F) negative control of Figure 4G; anti-CD68 (Alexa488, turquoise fluorescence) labeling the M1-like and M2-like macrophages.; anti-CD206 (Alexa568, red fluorescence) labeling the M2-like macrophages and satellite cells; nuclear staining with DAPI (blue fluorescence). Figure S4: In situ hybridization in the non-operated femur of the rat; (A) expression of β-actin mRNA (positive control); (B) negative control; (C) CD68 mRNA; (D) IL-1β mRNA; (E) CD163 mRNA; (F) IL-6 mRNA; (G) expanded view: high magnification image of the area within the red rectangle in image E; (H) expanded view: high magnification image of the area within the red rectangle in image F. Table S1: Oligos used for RT-qPCR: FW (forward primer); RV (reverse primer). Table S2. Oligos are used for in situ hybridization experiments: T3 (T3 promoter); T7 (T7 promoter); FW (forward primer); and RV (reverse primer).
