# Supplementary material for: Macrophages Characterization in an Injured Bone Tissue
Source: Biomedicines. 2022 Jun 11;10(6):1385. doi: 10.3390/biomedicines10061385 (PMC9219779; doi:10.3390/biomedicines10061385)
Supplement: Supplementary file 1 [file biomedicines-10-01385-s001.zip › biomedicines-1765346-supplementary.pdf]

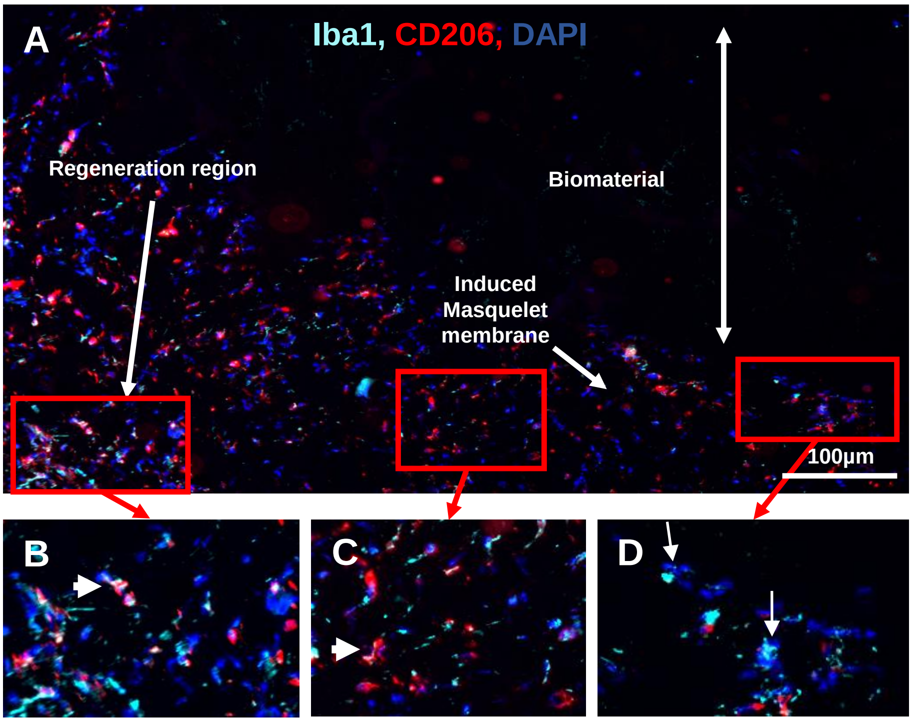

Figure S1. Identification of M1-like and M2-like macrophages in the rat femurs. (**A-D**) Immunolabeling with anti-Iba1 and CD206 antibodies of the operated femurs. (**B,C,D**) Expanded view: high magnification image of the area within the red rectangle in image A. Anti-Iba1 (Alexa488, turquoise fluorescence), labeling the M1 and M2 macrophages. Anti-CD206 (Alexa568, red fluorescence) labeling the M2 macrophages and satellite cells. Nuclear staining with DAPI (blue fluorescence). Thin arrow: M1-like macrophages; thick arrow: M2-like macrophages.

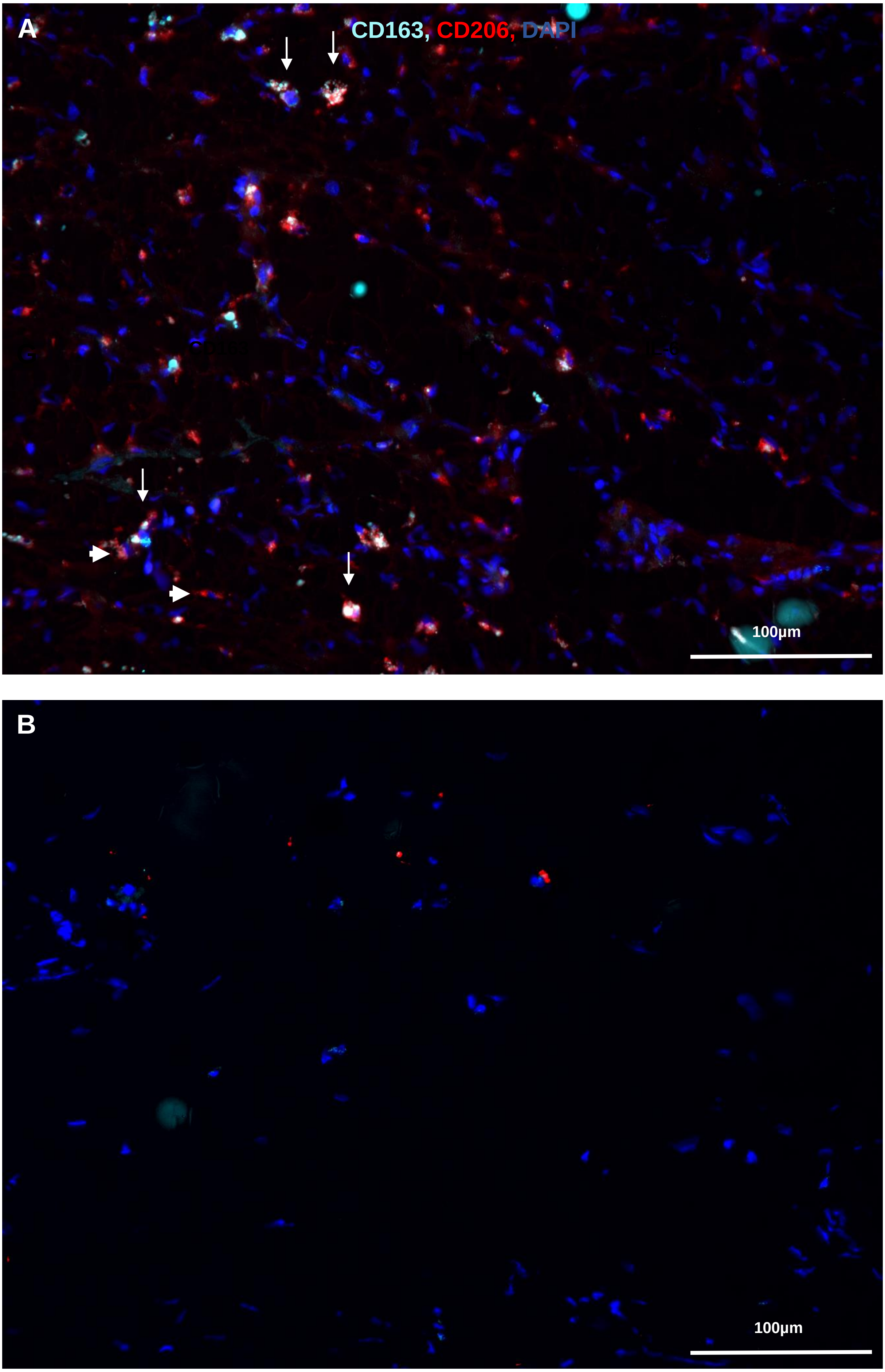

Figure S2. Identification of M2-like macrophages and satellite cells in the rat femurs. (**A**) Immunolabeling with anti-CD163 and CD206 antibodies of the operated femurs. (**B**) Negative control. Anti-CD163 (Alexa488, turquoise fluorescence), labeling the M2-like macrophages. Anti-CD206 (Alexa568, red fluorescence) labeling the M2-like macrophages and satellite cells. Nuclear staining with DAPI (blue fluorescence). Thin arrow: M2-like macrophages; thick arrow: satellite cells.

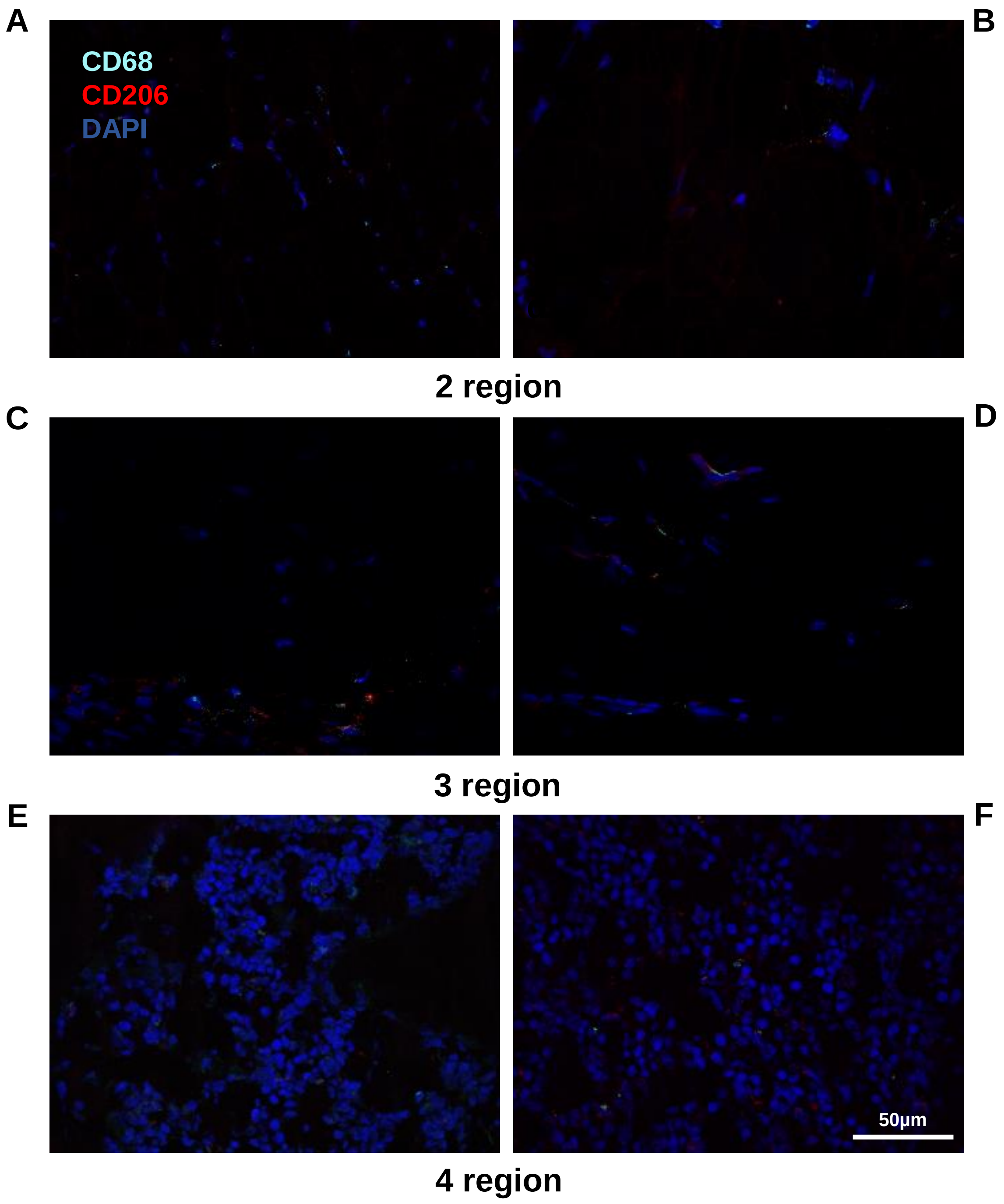

Figure S3. Identification of macrophages M1-like and M2-like in the femurs of the rat. (**A**) Negative control of Figure 4B; (**B**) Negative control of Figure 4C; (**C**) Negative control of Figure 4D; (**D**) Negative control of Figure 4E; (**E**) Negative control of Figure 4F; (**F**) Negative control of Figure 4G. Anti-CD68 (Alexa488, turquoise fluorescence) labeling the M1-like and M2-like macrophages. Anti-CD206 (Alexa568, red fluorescence) labeling the M2-like macrophages and satellite cells. Nuclear staining with DAPI (blue fluorescence).

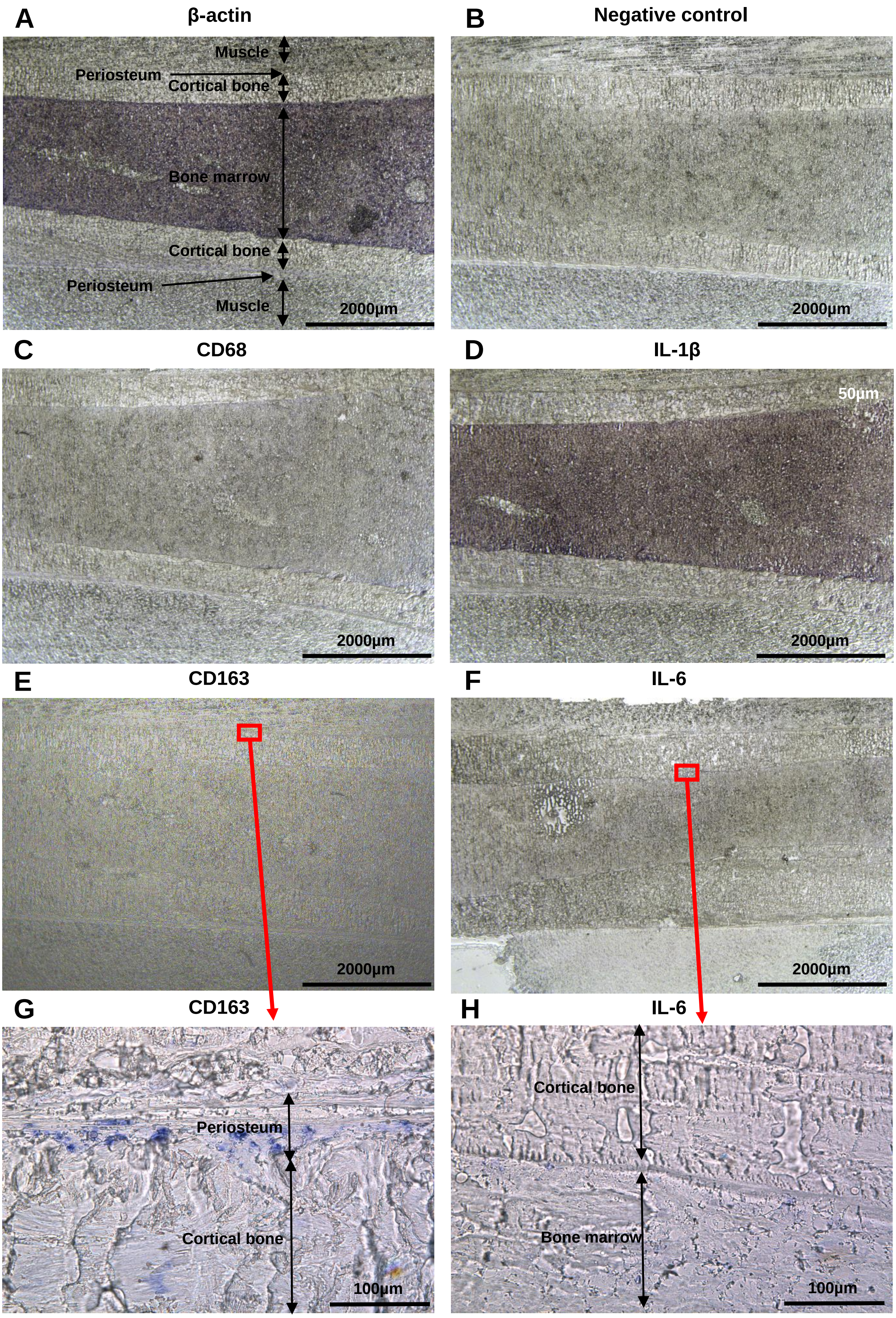

Figure S4. *In situ* hybridization in the non-operated femur of the rat. (**A**) Expression of β-actin mRNA (positive control); (**B**) negative control; (**C**) CD68 mRNA; (**D**) IL-1β mRNA; (**E**) CD163 mRNA; (**F**) IL-6 mRNA. (**G**) Expanded view: high magnification image of the area within the red rectangle in image E. (**H**) Expanded view: high magnification image of the area within the red rectangle in image F.

Oligo name	Gene accession number	Oligo sequence
RT_Rat_CD68_FW	XM_213372.3	CAATTCACCTGGACCTGCTCTC
RT_Rat_CD68_RV	XM_213372.3	AAGAGAAGCATGGCCCGAAG
RT_Rat_CD163_FW	XM_006237352.2	CCTTCTCATTGCCTTCCTCTTGTG
RT_Rat_CD163_RV	XM_006237352.2	TTCCGAGGATTTCAGCAAGTC
RT_Rat_CD206_FW	NM_001106123	TGGTCATCGTGGTCCTTCTGATTG
RT_Rat_CD206_RV	NM_001106123	GATCTTTCGTGTCACTTGTTCCAG
RT_Rat_actin_FW	NM_031144.3	CCTGGAGAAGAGCTATGAGCTG
RT_Rat_actin_RV	NM_031144.3	CAGGATTCCATACCCAGGAAGG
RT_Rib_prot_FW	FQ 211152.1	CTAAAATCTCCAGAGGTACCATTG
RT_Rib_prot_RV	FQ 211152.1	TCCCACCTTGTCTCCAGTCTTTATC
RT_Rat_IL-1β_FW	NM_031512.1	TCTTTGAAGAAGAGCCCGTCC
RT_Rat_IL-1β_RV	NM_031512.1	AGCTCACATGGGTCAGACAG
RT_Rat_IL-6_FW	NM_012589.1	GAGGATACCACCCACAACAGACC
RT_Rat_IL-6_RV	NM_012589.1	AGTGCATCATCGCTGTTCATACAA
RT_Rat_TNF_FW	NM_012675.3	CATCTTCTCAAAACTCGAGTGACAA
RT_Rat_TNF_RV	NM_012675.3	TGGGAGTAGATAAGGTACAGCCC
RT_Rat_IL-RA_FW	XM_006233636.3	TCGGAATGTGTTCTTGGGCATC
RT_Rat_IL-RA_RV	XM_006233636.3	TCGGAGCGGATGAAGGTAAAG
RT_Rat_IL-4_FW	NM_201270.1	CTTACGGCAACAAGGAACACC
RT_Rat_IL-4_RV	NM_201270.1	TGAGTTCAGACCGCTGACAC
RT_Rat_IL-10_FW	NM_012854.2	GCAGTGGAGCAGGTGAAGAATG
RT_Rat_IL-10_RV	NM_012854.2	TGAGTGTCACGTAGGCTTCTATGC
RT_Rat_TGF-β1_FW	NM_021578.2	CCGCAACAACGCAATCTATGAC
RT_Rat_TGF-β1_RV	NM_021578.2	CGTGTTGCTCCACAGTTGACTTG
RT_Rat_TGF-β2_FW	XM_006250448.3	GGCTTCACCACAAAGACAGGAAC
RT_Rat_TGF-β2_RV	XM_006250448.3	CCTCCAGCTCTTGGCTCTTATTTG
RT_Rat_TGF-β3_FW	NM_013174.2	AGGAGTGGACAACGAAGATGAC
RT_Rat_TGF-β3_RV	NM_013174.2	CAGTCGGTGTGGAGGAATCATC

Table S1. Oligos used for RT-qPCR. FW (forward primer), RV (reverse primer).

**Oligo name**	**Gene accession number**	**Oligo sequence**
T3_promoter_primer		GCTACTTGCTGAATTAACCCTCACTAAAGGGA
T7_promoter_primer		AGTCGAATGTAATACGACTCACTATAGGGC
DIG_Rat_actin_FW_T3	NM_031144.3	TGAATTAACCCTCACTAAAGGGACCATTGAACACGGCATTGTC
DIG_Rat_actin_RV_T7	NM_031144.3	TGTAATACGACTCACTATAGGGCAAACGCAGCTCAGTAACAGTCC
DIG_Rat_CD68_FW_T3	XM_213372.3	TGAATTAACCCTCACTAAAGGGATGACCTTGCTGGTACTGCTTGTAG
DIG_Rat_CD68_RV_T7	XM_213372.3	TGTAATACGACTCACTATAGGGCTACAGAGTGGACTGGAGCAAATG
DIG_Rat_CD163_FW_T3	XM_006237352.2	TGAATTAACCCTCACTAAAGGGATCTCTGAGGCTGACCAATGAAG
DIG_Rat_CD163_RV_T7	XM_006237352.2	TGTAATACGACTCACTATAGGGCAGATGTAGCTGTGGTCATCC
DIG_Rat_IL-1β_FW_T3	NM_031512.1	TGAATTAACCCTCACTAAAGGGAATGACCTGTTCTTTGAGGCTGAC
DIG_Rat_IL-1β_RV_T7	NM_031512.1	TGTAATACGACTCACTATAGGGCTCAATTCATCCCATACACACGGAC
DIG_Rat_IL-6_FW_T3	NM_012589.1	TGAATTAACCCTCACTAAAGGGACACCAGGAACGAAAGTCAACTC
DIG_Rat_IL-6_RV_T7	NM_012589.1	TGTAATACGACTCACTATAGGGCAGAAACCATCTGGCTAGGTAAGAG
HCR_rat_CD163_revcom_fragment1	XM_006237352.2	GAGGAGGGCAGCAAACGGGAAGAGTCTTCCTTTACGATATTGCGCAGCGACCACCTCCACCTACCAAGCGGAGTTGACCACTTGCTATGCAATATAGCATTCTTTCTTGAGGAGGGCAGCAAACGGGAAGAG
HCR_rat_CD163_revcom_fragment2	XM_006237352.2	GAGGAGGGCAGCAAACGGGAAGAGTCTTCCTTTACGATATTGTCAGCCTCAGAGACATGAACTCCGAGCAGACAACACCTGCATCTTCCTTATATAGCATTCTTTCTTGAGGAGGGCAGCAAACGGGAAGAG
HCR_rat_CD163_revcom_fragment3	XM_006237352.2	GAGGAGGGCAGCAAACGGGAAGAGTCTTCCTTTACGATATTCTGTAGTCTTATTTTGTCGTCACAGACAATCCAGGACTCCTGGGAGGGGCATATAGCATTCTTTCTTGAGGAGGGCAGCAAACGGGAAGAG
HCR_rat_CD163_revcom_fragment4	XM_006237352.2	GAGGAGGGCAGCAAACGGGAAGAGTCTTCCTTTACGATATTCACAAACCAGGAGTGCAGTGAGGGTTGAATGACCTGTGCCATGCTGTGATATATAGCATTCTTTCTTGAGGAGGGCAGCAAACGGGAAGAG
HCR_rat_CD163_revcom_fragment5	XM_006237352.2	GAGGAGGGCAGCAAACGGGAAGAGTCTTCCTTTACGATATTCATTAAAATCATACGAATCATTGGAATTTTCCGAGGATTTCAGCAAGTCCATATAGCATTCTTTCTTGAGGAGGGCAGCAAACGGGAAGAG
HCR_rat_ IL-1β_revcom_fragment1	NM_031512.1	CCTCGTAAATCCTCATCAATCATCCAGTAAACCGCCAAAAAACTCCTCACTGTCGAAAGCTGCTATTTCACAGTTGAGTTCAGGGACAGTTGAAAAAAAGCTCAGTCCATCCTCGTAAATCCTCATCAATCATC
HCR_rat_ IL-1β_revcom_fragment2	NM_031512.1	CCTCGTAAATCCTCATCAATCATCCAGTAAACCGCCAAAAAATGGATGCTCTCATCTGGACAGCCCAAGTCAAGGGCTTGGAAGCAATCCTTAAAAAAAGCTCAGTCCATCCTCGTAAATCCTCATCAATCATC
HCR_rat_ IL-1β_revcom_fragment3	NM_031512.1	CCTCGTAAATCCTCATCAATCATCCAGTAAACCGCCAAAAAAGTCATCATCCCACGAGTCACAGAGGACGGGCTCTTCTTCAAAGATGAAGGAAAAAAAGCTCAGTCCATCCTCGTAAATCCTCATCAATCATC
HCR_rat_ IL-1β_revcom_fragment4	NM_031512.1	CCTCGTAAATCCTCATCAATCATCCAGTAAACCGCCAAAAAACTTGTACAAAGCTCATGGAGAATACCACTTGTTGG

		CTTATGTTCTGTCCAAAAAAAAGCTCAGTCCATCCTCGT AAATCCTCATCAATCATC
HCR_rat_ IL-1β_revcom_fragment5	NM_031512.1	CCTCGTAAATCCTCATCAATCATCCAGTAAACCGCCAAA AAAGTATTGTTTGGGATCCACACTCTCCAGCTGCAGGGT GGGTGTGCCGTCTTAAAAAAAGCTCAGTCCATCCTCGTA AATCCTCATCAATCATC
HCR_rat_ IL-6_revcom_fragment1	NM_012589.1	CTCACTCCCAATCTCTATCTACCCTACAAATCCAATAAA AAAGTCCCAAGAAGGCAACTGGCTGGAAGTCTCTTGCG GAGAGAAACTTCATATTTTCACTTCATATCACTCACTCC CAATCTCTATCTACCC
HCR_rat_ IL-6_revcom_fragment2	NM_012589.1	CTCACTCCCAATCTCTATCTACCCTACAAATCCAATAAA AAAATTGCCATTGCACAACTCTTTTCTCATTTCCAAGAT CTCCCTGAGAACAATTTTCACTTCATATCACTCACTCCC AATCTCTATCTACCC
HCR_rat_ IL-6_revcom_fragment3	NM_012589.1	CTCACTCCCAATCTCTATCTACCCTACAAATCCAATAAA AAGGAACTCCAGAAGACCAGAGCAGATTTTCAATAGGC AAATTTCCTGGTTAATTTTCACTTCATATCACTCACTCCC AATCTCTATCTACCC
HCR_rat_ IL-6_revcom_fragment4	NM_012589.1	CTCACTCCCAATCTCTATCTACCCTACAAATCCAATAAA AATGGAAGTTGGGGTAGGAAGGACTATTTTATATGAGT CTTTTATCTCTTGTATTTTCACTTCATATCACTCACTCCC AATCTCTATCTACCC
HCR_rat_ IL-6_revcom_fragment5	NM_012589.1	CTCACTCCCAATCTCTATCTACCCTACAAATCCAATAAA AAATGCTTAGGCATAGCACACTAGGTTTGCCGAGTAGA CCTCATAGTGACCTATTTTCACTTCATATCACTCACTCCC AATCTCTATCTACCC

Table S2. Oligos used for *in situ* hybridization experiments. T3 (T3 promoter), T7 (T7 promoter), FW (forward primer), RV (reverse primer).
